# Validation on Method of Measuring the Knee Joint Force Sense and Application in Patients With Knee Osteoarthritis

**DOI:** 10.1155/abb/8851877

**Published:** 2025-07-03

**Authors:** Zhixin Jin, Jingnan Shi, Haohua Zhang, Xinglong Zhou, Kuan Zhang, Songhua Yan

**Affiliations:** ^1^Beijing Sport University, Beijing 100084, China; ^2^School of Biomedical Engineering, Capital Medical University, Beijing 100069, China; ^3^Department of Orthopedics, Beijing Jishuitan Hospital, Capital Medical University, Beijing 100035, China

**Keywords:** force sense, knee osteoarthritis, proprioception

## Abstract

**Background:** Force sense reflects the efferent activity capacity of proprioception. Currently, methods for measuring force sense in the knee joint is lack. This study aims to validate a constructed test system and to explore the characteristics of force sense in patients with knee osteoarthritis (KOA).

**Methods:** Seventy-five subjects were recruited: 30 for verifying the reliability of test system and exploring the impact of body mass index (BMI) and the striking force on force sense; 20 healthy adults and 25 KOA patients for the method application. The force sense test system comprised a self-designed force application apparatus and a wireless surface electromyography (sEMG) device (DELSYS Inc, USA). The reflex contraction latency of muscles was considered as the force sense. The intraclass correlation coefficient (ICC) was used to verify the reliability.

**Results:** The reflex contraction latencies of biceps femoris (BF) was the shortest and ICC in the two tests is 0.950 (*p* < 0.001). No significant differences in force sense were found between different BMI groups (*p*=0.065), and there was no notable interaction between BMI and striking force (*p*=0.283). A significant difference in force sense was observed between different striking forces (*p* < 0.001). There were no significant differences in force sense of bilateral sides between healthy people and KOA patients (*p*=0.126, *p*=0.315).

**Conclusion:** The force sense testing method established in this study is applicable for measuring knee joint force sense. BMI did not affect knee joint force sense but striking force did, and subjects with different BMI chose the same striking force; KOA did not influence the force sense of knee joint.

## 1. Introduction

Proprioception consists of three components: joint position sense, pertaining to static awareness of joint position; joint kinesthesia, which refers to the detection of movement and acceleration; and joint force sense, in terms of the reflex contraction latency of muscle surrounding the joint. The former two aspects are indicative of proprioception's afferent aspects of proprioception, whereas joint force sense shows the efferent activity capacity associated with reflex response and regulation of muscle stiffness [1, 2]. However, the majority of researches on knee joint proprioception have primarily centered on its afferent activity capabilities, with limited focus on its efferent abilities [3], partly due to the absence of a standardized apparatus and precise measurement. Muscle contraction and the regulation of muscle tone protect the joint actively. For both low and high loads, the force sense receptors will stimulate the tendons to release electrical signals, providing proprioceptive information for joint movement. It plays a crucial role in maintaining the functional stability of the joint [4–6]. For measurement of proprioception, joint position sense and kinesthesia already have the widely-accepted testing methods, which are joint position reproduction (JPR) [7] and threshold to detect passive motion (TDPM) [8], respectively. The measurement of joint force sense includes two methods, such as target force reproduction or reflecting joint force sense through muscle reflex response latency. The muscle reflex response latency is obtained by applying mechanical or electrical stimulation to the area around the knee joint and recording changes in surface electromyography (sEMG) or cortical potentials. Most studies use external perturbations to induce potential changes, and the muscle reflex response ability is assessed through the latency of the potentials [9, 10].

Knee osteoarthritis (KOA) is a degenerative condition that leads to the deterioration of joint cartilage and bone, presenting symptoms, such as joint pain, stiffness, and swelling [11]. Additionally, extensive research has shown that KOA has a considerable impact on proprioception in the knee joint [12, 13]. KOA patients have the poorer stability of the knee joint, thereby impacting the lower limb function and more than 60% of KOA patients reported knee instability and the phenomenon of “giving way” [14–16]. There are already some studies described gait changes [17] and more changes in the muscle activation patterns of key lower limb muscles in KOA patients due to their instability in the knee joint [18, 19]. However, it is unknown whether the knee joint force sense changed, thereby affecting their joint stability in KOA patients.

Target force reproduction may be the most direct method for testing knee joint force sense. However, KOA patients often suffer varying degrees of pain, which is highly subjective and may affect the results, or they could even not complete the test. The approach of assessing force sense through muscle reflex contraction latency was predominantly employed in the investigation of functional ankle instability (FAI) [20, 21] and the wrist [22], with limited application to knee joints. Bread et al. [2, 23] used a metal frame to stabilize the legs of patients with anterior cruciate ligament deficiency (ACLD) and applied a piston to strike the posterior popliteal fossa with a force of 100 N. They measured the activity of the hamstring muscles using electromyography and assessed the acceleration of the leg after the impact with an accelerometer, calculating the reflex hamstring contraction latency (RHCL) to reflect the patients' joint force sense. However, their pneumatic device was easily affected by external environmental factors and applied only a single load. The time measured for tibial displacement was slightly delayed compared to the moment of knee joint stimulation, which reduced measurement accuracy, and the muscles responded to maintain balance when a perturbation was suddenly applied, and the response would change with the speed of perturbation increased [24]. Because the speed of the joint's passive movement increases with the velocity of the perturbation increases. Similarly, the passive movement speed of the joint increases as the striking force increases. Therefore, the choice of strike force may affect the results of the knee joint force sense. In addition, proprioception can be influenced by obesity [25, 26], but the studies only focus on the differences in position sense and kinesthesia among individuals with different body mass index (BMI). It is unclear whether BMI affects joint force sense.

Consequently, our hypothesis is that different levels of striking force and obesity have an impact on knee joint force sense. Additionally, KOA will influence knee joint force sense. This study aims to establish a methodology for measuring knee joint force sense and to investigate whether striking force and obesity affect it. Furthermore, we aim to reveal the characteristics of the force sense in KOA patients compared to healthy individuals using this validated method.

## 2. Materials and Methods

### 2.1. Validation for the Measurement of the Knee Joint Force Sense

The reliability of the devised method for measuring knee joint force sense was rigorously examined, the potential influences of BMI and striking force on it were considered concurrently.

A self-designed exerting force apparatus and a wireless surface electromyography (sEMG) system (Trigno Wireless Systems, DELSYS Inc., USA) were used to measure the force sense of the knee joint (patent number: ZL 2020 1 1268463.8) [27]. The sampling frequency of the sEMG system is 2000 Hz.

The device used to exert force on the knee joint was capable of applying various force interferences to the proximal tibia region located on the posterior aspect of the knee and it mainly used the principle of simple pendulum. To meet different participants, the height of the pendulum could be adjusted, ensuring a consistent impact location throughout the trials. The range of striking forces ranged from 50 to 200 N, with a designed interval of 10 N between each force level. The pendulum was raised to the specified height according to the selected force level [28].

Once the system was thoroughly prepared, activation of the release button initiated the pendulum's descent, culminating in the strike. Simultaneously, an indicator light illuminated, confirming that the impact had been executed smoothly and successfully.

The intraclass correlation coefficient (ICC) serves as the primary metric for assessing retest reliability, with values exceeding 0.90, ranging from 0.75 to 0.90, from 0.50 to 0.75, and below 0.50, indicative of excellent, good, moderate, and poor reliability, respectively [29].

Thirty healthy male individuals with different BMI who voluntarily participated in the experiment were recruited. Individuals were included if they were healthy and over 18 years old; had a right dominant limb, which was defined as the limb with which they would kick a ball; did not have lower limb injuries, lower limb surgeries, nervous system disease, peripheral nerve injury or other metabolic diseases; or did not have visual, vestibular disorders or experience of professional sports training. Using GPower_3.1.9.7 for power analysis, it was found that a sample size of 30 participants would provide 85% power to detect a significant difference in the knee joint force sense among normal, overweight and obese groups. According to their BMI (weight/height^2^, kg/m^2^), they were divided into three groups: the normal group (18.5 ≤ BMI < 24), the overweight group (24.0 ≤ BMI <28) and the obese group (BMI ≥ 28), with 10 individuals in each group. The basic information of the subjects is shown in Table 1.

All participants voluntarily signed an informed consent form prior to their involvement, ensuring their understanding of the study's purpose, procedures, and potential risks. The study was approved by the ethics committee (Ethical batch number: 2021SY093).

Two consecutive experiments were conducted, each with a standardized striking force of 100 N. The muscle reflex contraction latencies of the biceps femoris (BF) and semitendinosus (ST) were recorded in both experiments, and the corresponding ICCs were subsequently calculated.

**Figure 1:**
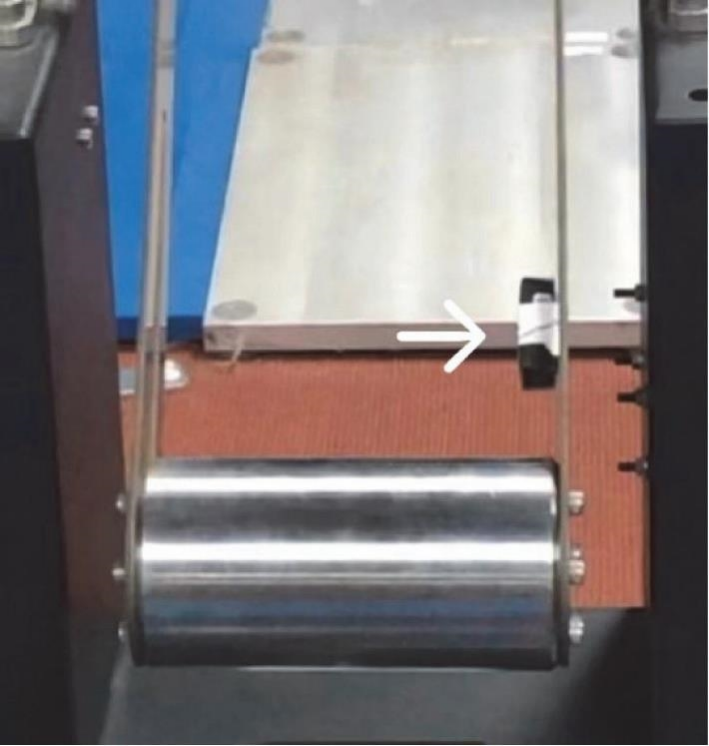
Fixed position of an accelerometer (→ refers to the accelerometer).

To minimize skin impedance, the areas of electrode placement were shaved, rubbed, and subsequently cleaned with isopropyl alcohol swabs. The EMG electrodes placements followed the surface electromyography for the noninvasive assessment of muscles (SENIAM) guidelines and placed on the most prominent part of the muscle belly. An accelerometer-equipped electrode was affixed to the pendulum (Figure 1) to determine the instant of pendulum impact, allowing for the synchronous collection of EMG and acceleration signals.

Following electrode placement, participants stood while the platform height of the force-exerting device was adjusted to match their individual stature, ensuring that the pendulum struck the posterior popliteal fossa.

**Figure 2:**
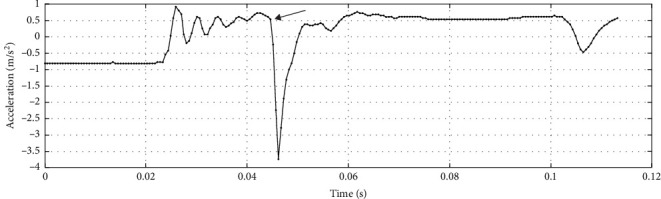
Moment of sudden acceleration change (→ refers to change-point).

A fixed striking force of 100 N was utilized in previous studies [2], but in fact the optimal force remains uncertain. In our study, we selected a range of striking forces (50, 70, 100, 120, and 150 N) to explore this variable. A single-blind method was employed in which the individuals were unaware of which group they had been assigned to and the order of striking force application, while the researchers were informed. To prevent the reaction of individuals to smaller forces from being interfered with after applying larger forces, we uniformly applied the striking forces in ascending order. After each test, participants rested for 2 min to mitigate fatigue effects. Each force level was tested three times, and the muscle around the knee exhibiting the shortest reflex response latency, based on the order of muscle response, was chosen for further analysis. The variability of muscle reflex response latency was calculated to ascertain the most appropriate striking force for our study. The data were processed using EMGworks Analysis_4.7.3.0, which was designed for use with the DELESYS wireless sEMG system. The acceleration data in the swing direction of the hammer were selected, and the time at which the acceleration suddenly changed was defined as the striking time of the pendulum (Figure 2).

After removing an offset from the EMG signal, filtering consisted of a fourth-order Butterworth band-pass filter with a cut-off frequency range of 20–500 Hz. And the absolute values of signals were computed and calculated its root mean square (RMS) with a 30 ms sliding window length and 20 ms overlap. The abrupt change in RMS indicated the onset of muscle reflex contraction. Subsequently, muscle contraction latency was determined by subtracting the pendulum striking time from the time of muscle activation [2]. To evaluate knee joint force sense, the average latency derived from three trials at the same force level was considered.

The random effects model was employed to analyze the ICC between the two measurement outcomes. To discern the order of reflex contraction among the five muscles, descriptive statistics were applied. The normality of the data was verified using the Shapiro–Wilk test, revealing approximate conformity with a normal distribution across the three datasets. There were five levels of striking force as within-subject variables and three different BMI as between-subject variables. Therefore, a two-way repeated measures analysis of variance (ANOVA) was utilized to examine the influence of BMI and striking force on knee joint force sense, as well as to test whether there is an interaction between these two variables. The statistical significance was set at *p* < 0.05. All analyses were conducted using SPSS 23.0 software (SPSS Inc., Chicago, IL, USA).

The intra-group correlation analysis was conducted on the reflex contraction latencies of BF (ICC = 0.950, *p* < 0.001) and ST (ICC = 0.942, *p* < 0.001) in the two tests. The reflex contraction latency of BF (30.69 ms) and ST (37.64 ms) were compared and the reflex contraction latency for BF was the shortest and thus selected as a parameter to reflect the knee joint force sense. The reflex response latency of BF under different BMI and striking forces are shown in Table 2.

The study revealed a statistically significant difference in the reflex contraction latency of BF among varying striking forces (*p* < 0.001). Conversely, no significant difference (*p*=0.198) was observed in this latency among the three groups considered. Furthermore, the analysis failed to demonstrate a notable interaction between BMI and striking force (*p*=0.101).

### 2.2. Knee Joint Force Sense in Patients With KOA

#### 2.2.1. Participants

Twenty-five KOA patients with indications for knee arthroplasty from the Orthopedic Department at Beijing Jishuitan Hospital, China were recruited as the test group. Inclusion criteria encompassed individuals aged 50–70 years, with KOA and surgical indications, capable of comprehending basic instructions and cooperating with experiments, and volunteering for the study. Exclusion criteria were rheumatic diseases affecting the knee joint, visual or vestibular impairments that could disrupt sensory input, history of neurological disorders or peripheral nerve injuries (e.g., Parkinson's disease, Alzheimer's disease), knee injuries, cerebrovascular accidents, knee flexion contractures exceeding 15°, had any other issues related to the lower extremities, or taking medication affecting neuromuscular function.

Twenty middle-aged, healthy volunteers without apparent knee disease were selected as the control group. Inclusion criteria were: age matching the experimental group (50–70 years), right leg dominance, no history of knee injury, surgery, heart infarction, brain infarction, cardiovascular disease, diabetes, thyroid, adrenal, neurological disorders, peripheral nerve injuries, or other metabolic conditions; absence of visual or vestibular impairments; and no professional sports training experience. Using GPower_3.1.9.7 for power analysis, it was found that a sample size of 45 participants (25 KOA patients and 20 healthy volunteers) would provide 90% power to detect a significant difference in the knee joint force sense between KOA patients and healthy individuals.

Prior to the experiment, participants' basic information, including name, age, height, weight, and BMI, was recorded (Table 3). All subjects provided informed consent and were informed of the study's protocol and precautions, adhering to the experimental requirements throughout the testing procedure.

Although a significant difference in BMI and age exists between the two groups, we have validated that BMI does not affect knee joint force sense. Some studies have reported differences in proprioception between young and elderly individuals [30–33]. However, our research has shown that there is no difference in proprioception between individuals aged 50 and those aged 60 [34].

#### 2.2.2. Methods

The knee joint force sense of KOA patients was evaluated using the above validated methods and data processing techniques described above. Given the clinical feasibility, the striking forces during the experiment were limited to 50, 100, and 150 N. Following the interference, the knee joint was positioned in passive flexion.

The coefficient of variation (CV), a standardized measure of dispersion, was employed to identify the optimal striking force. A lower CV value signifies reduced dispersion, indicative of a more stable indicator. In this study, CV was calculated for muscle reflex contraction latency to determine the most suitable striking force.

For statistical analysis, SPSS23.0 and Microsoft Excel software were employed. The Shapiro–Wilk test was used to verify the normality of the data distributions. Normally distributed data were presented as mean ± standard deviation, while abnormally distributed data were described by median (upper quartile and lower quartile). A repeated measure ANOVA was used to test the differences between KOA patients and healthy individuals, between the bilateral limbs, and the interaction between group and side. And the results were corrected by the Bonferroni. The significance level was set at *p* < 0.05.

During data analysis, it was observed that signals from KOA patients exhibited disarray under a striking force of 50 N, and the muscle activation was unobservable in some KOA patients. Therefore, the 50N may be insufficient to elicit the muscle reflex contraction latency in KOA patients, potentially introducing bias into the findings. Consequently, this force level was deemed inappropriate for KOA subjects. To ensure the integrity of the study, only data obtained at striking forces of 100 and 150 N were considered for further analysis and discussion.

## 3. Results

**Figure 3:**
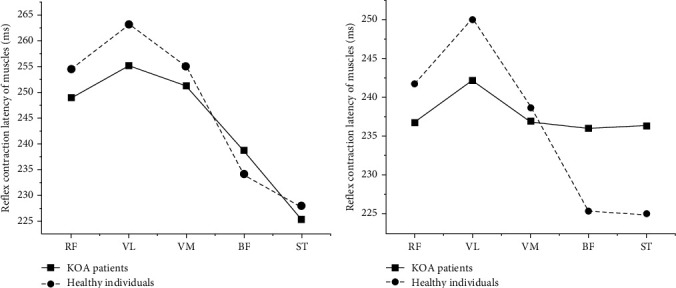
The reflex contraction latency of muscles (A) indicates the reflex contraction latency of muscles at 100 N; (B) indicates the reflex contraction latency of muscles at 150 N. BF, biceps femoris; RF, rectus femoris; ST, semitendinosus; VL, vastus lateralis; VM, vastus medialis.

The reflex contraction latency of BF (233.98 and 225.42 ms) and ST (227.81 and 224.93 ms) on the right side of healthy people and the reflex contraction latencies of BF (238.62 and 236.40 ms) and ST (225.28 and 236.00 ms) on the affected side of KOA patients at 100 and 150 N were calculated (Figure 3). The latency of ST was the shortest and remained consistent across healthy individuals and KOA patients at both 100 and 150 N striking forces and was chosen as an indicator of knee joint force sense.

The CVs for the reflex contraction latency of ST were computed separately for the right side of healthy participants (CV_100 N_ = 0.095 and CV_150 N_ = 0.076) and the affected side of KOA patients (CV_100 N_ = 0.153 and CV_150 N_ = 0.097), under both 100 and 150 N striking forces. At the striking force of 150 N, the CVs for the reflex contraction latency of ST in both healthy people and KOA patients exhibited lower values compared to those at 100 N, indicating reduced dispersion. Consequently, 150 N was chosen as the optimal striking force for this study.

The comparative results of reflex contraction latency for bilateral ST between the two groups are shown in Table 4.

There were no significant differences between healthy people and KOA patients in reflex contraction latencies of bilateral ST (*p* > 0.05). Also, there were no significant differences in reflex contraction latency of ST between bilateral sides for both healthy people and KOA patients (*p* > 0.05).

## 4. Discussion

The reliability of the method utilized to assess knee joint force sense has been thoroughly verified. This study delved into the variations in muscle reflex contraction latency among three distinct groups of healthy participants, each characterized by a different BMI, in response to varying striking forces. The findings revealed that BMI did not exert a significant impact on knee joint force sense, which does not support our hypothesis. In contrast, the applied striking force emerged as a crucial determinant, supporting our hypothesis. Furthermore, BMI and striking force were identified as independent variables influencing knee joint force sense, with no observed interaction between them. In the sequence of muscle activation among both healthy individuals and KOA patients, ST was the first to activate. Consequently, its reflex contraction latency was adopted as a parameter to evaluate knee joint force sense. Given the striking force's potential impact on knee joint force sense, CV was employed to determine the optimal striking force for this study. Our findings indicated that a striking force of 150 N yielded the smallest CV for the reflex contraction latency of ST. Therefore, when using the reflex contraction latency of ST as a parameter for knee joint force sense, 150 N was identified as the most suitable striking force to investigate its effect in KOA patients.

Both Çiftçi [25] and Wang et al. [26] showed that BMI affected position sense and kinesthesia respectively, which were different from the findings of this study. Proprioception is originated by the brain processing afferent information from proprioceptors and mechanoreceptors [35]. Proprioceptors include muscle spindles, processing information about muscle length and the rate of stretch, and Golgi tendon organs, transmitting information about tension and the resulting contraction force [35, 36]. Mechanoreceptors include Pacinian corpuscles, Ruffini endings, Merkel cells, and Meissner corpuscles, which are located in the skin, ligaments, and joint capsules [35, 36]. Increased body weight leads to increased load on the knee joints and long-term exposure to excessive and abnormal joint loads may cause damage to the surrounding tissues of the joints [37, 38]. Obesity increases the load on the knee joint and influences the mechanoreceptors located in the skin, ligaments, and joint capsules, while its effect on proprioceptors is lower [26]. Therefore, BMI is not a factor to affect the knee joint force sense in this study.

The present study revealed that KOA exerts minimal impact on knee joint force sense, which is not same as our hypothesis. Related study remains scarce. Given this scarcity, our investigation drew parallels from previous findings on patients with ACLD. Jennings' study [39] concurred with our observations, reporting no discernible difference in force sense between affected and unaffected ACLD limbs. In contrast, Bread's research [23] identified a marked prolongation in RHCL on the affected ACLD side, possibly attributed to variations in the applied striking force. For research on ankle joint proprioception, it has been found that in patients with FAI, the latency of peroneal muscle response is prolonged during sudden ankle inversion [40] and it suggests that a longer muscle reflex response latency may indicate joint instability.

Force sense reflects the efferent ability of proprioception, and the neural feedback mechanism in joints and tendons provides an essential component for maintaining joint functional stability [41]. Muscle activation and joint mobility are regulated by the brain and spinal cord, which integrate external sensory inputs primarily through the central nervous system (CNS). Afferent information undergoes processing at three distinct motion control levels: spinal, brainstem, and cerebral centers [42–44]. The assessment of knee joint force sense primarily involves quantifying muscle activation latency during involuntary perturbations, serving as an index of spinal cord reflex pathways. Delayed reflexes may compromise joint stability, predisposing to degeneration. Especially our study found no significant differences in force sense between KOA patients and healthy individuals, suggesting that while KOA causes degenerative changes in the knee joint and receptor impairment, exerts minimal impact on protective spinal reflexes [45]. KOA patients exhibited the similar reflex response latency to those of healthy individuals may be explained by feedforward (preparatory) control [46]. Muscle tension is produced to prepare the joint for impact and to cope with unforeseen disturbances or unexpected events before each joint load. This is manifested as increased muscle activity and stiffness around the joint due to the joint preactivation [47, 48]. Additionally, this preactivation enhances the afferent activity of muscle spindles around the joint through alpha–gamma coactivation, allowing for faster detection of length changes and a faster, larger reflex response latency [49]. Therefore, KOA may only affect the feedback loop of neuromuscular control, while the equally important feedforward (preparatory) control remains unaffected.

The method established in this study for measuring knee joint force sense could provide clinicians with an effective tool to assess the efferent capabilities of proprioception in the knee joint. This will contribute to a more comprehensive evaluation of knee joint dysfunction and offer a better guidance for effective rehabilitation of patients. And KOA patients may benefit from enhanced proprioception training and neuromuscular training.

And there are some limitations in this study. Future research could increase the sample size to explore the potential differences within the KOA and healthy populations. The striking force affects knee joint force sense. While this study selected 150 N as the more appropriate force level among 100 and 150 N, we still do not know the optimal striking force for studying KOA patients.

## 5. Conclusion

Knee joint stability is very important for KOA patients and is influenced by proprioception. Our method effectively measured knee joint force sense in KOA patients, providing a tool for clinicians and researchers to assess proprioception comprehensively and guide rehabilitation strategies. We found that BMI did not significantly impact knee joint force sense, whereas striking force was a pivotal factor and future research could focus on determining the best striking force and combine the muscle co-contraction around the knee joint to explain the knee stability comprehensively. KOA did not significantly alter knee joint force sense but it helps us have a deeper understanding of the impact of KOA on proprioception.

## Figures and Tables

**Table 1:** Basic information of subjects in method validation.

Index	Normal group (*n* = 10)	Overweight group (*n* = 10)	Obese group (*n* = 10)	*p*
Age (years)	22.7 ± 1.00	22.40 ± 2.20	22.10 ± 1.97	0.781
Height (m)	1.77 ± 0.06	1.84 ± 0.05	1.81 ± 0.07	0.076
Weight (kg)	67.80 ± 4.43	87.22 ± 4.68	97.01 ± 10.03	<0.001*⁣*^*∗*^
BMI (kg/m^2^)	21.67 ± 1.16	25.80 ± 0.55	29.72 ± 1.90	<0.001*⁣*^*∗*^

*⁣* ^*∗*^Indicated significant difference at a level of 0.05.

**Table 2:** Comparison of reflex contraction latency of BF between three groups under different striking force (ms).

Parameters	50 N	70 N	100 N	120 N	150 N	*p*-Value	*F*	*η* ^*2*^
	M ± SD	M ± SD	M ± SD	M ± SD	M ± SD			
Normal	46.33 ± 24.25	31.67 ± 14.74	23.67 ± 16.94	17.67 ± 9.40	12.67 ± 8.32	—	—	—
Overweight	54.37 ± 21.67	40.33 ± 18.94	31.17 ± 17.32	26.00 ± 11.12	21.67 ± 11.22	—	—	—
Obesity	35.17 ± 23.66	39.67 ± 20.50	31.33 ± 16.14	24.00 ± 12.18	24.67 ± 15.11	—	—	—
*p*_1_	—	—	—	—	—	0.198	1.732	0.113
*p*_2_	—	—	—	—	—	<0.001*⁣*^*∗*^	21.70	0.446
*p*_3_	—	—	—	—	—	0.101	1.954	0.126

*Note*: *p*_1_ indicated the results of BMI main effect; *p*_2_ indicated the results of striking force main effect; *p*_3_ indicated the results of the interaction between BMI and striking force.

*⁣* ^*∗*^Indicated significant difference at a level of 0.05.

**Table 3:** Basic information of subjects.

Parameters	Age (years)	Height (m)	Weight (kg)	BMI (kg/m^2^)
Test group (*n* = 25)	61.92 ± 5.27	1.62 ± 0.08	71.64 ± 11.24	27.19 ± 3.86
Control group (*n* = 20)	55.15 ± 4.55	1.65 ± 0.08	68.20 ± 7.70	25.12 ± 2.77
*p*	0.001*⁣*^*∗*^	0.234	0.168	0.019*⁣*^*∗*^

*⁣* ^*∗*^Indicates significant difference at a level of 0.05.

**Table 4:** Reflex contraction latencies of bilateral ST (ms).

Parameters	Right/affected	Left/unaffected	*p*-Value	*F*	*η* ^*2*^
Healthy people (*n* = 20)	211.19 (210.68, 235.34)	232.36 (228.33, 244.82)	—	—	—
KOA patients (*n* = 25)	231.58 (218.34, 242.48)	229.24 (217.30, 243.52)	—	—	—
*p*_1_	—	—	0.235	1.458	0.037
*p*_2_	—	—	0.402	0.717	0.019
*p*_3_	—	—	0.508	0.447	0.012

*Note*: *p*_1_ indicated the comparison results between healthy people and KOA patients; *p*_2_ indicated the comparison results between the bilateral lower limbs both in healthy people and KOA patients; *p*_3_ indicated the results of the interaction between KOA and bilateral lower limbs.

## Data Availability

Research data are not shared.
